# MOTOR IMAGERY ENHANCES EARLY REHABILITATION IN PATIENTS WITH HIGH KINESIOPHOBIA AFTER TOTAL KNEE ARTHROPLASTY: A RANDOMIZED CONTROLLED TRIAL

**DOI:** 10.2340/jrm.v57.43688

**Published:** 2025-11-25

**Authors:** Jejeong LEE, Minjeong KIM, Yongwoo LEE

**Affiliations:** 1 Department of Physical Therapy, Graduate School, Sahmyook University, Seoul, Republic of Korea; 2 Department of Physical Therapy, College of Health and Welfare, Sahmyook University, Republic of Korea

**Keywords:** motor imagery, total knee arthroplasty, kinesiophobia, psychological factors, postoperative rehabilitation

## Abstract

**Objective:**

To investigate the effects of motor imagery on psychological and physical outcomes during acute-phase rehabilitation following total knee arthroplasty.

**Design:**

A single-blind, randomized controlled trial.

**Subjects/Patients:**

Forty patients who underwent total knee arthroplasty and scored ≥37 on the Tampa Scale of Kinesiophobia were enrolled and randomly allocated to either a motor imagery group or a control group.

**Methods:**

Both groups received the same 2-week standard rehabilitation programme beginning within 48 h post-surgery. The motor imagery group additionally performed structured motor imagery sessions prior to exercise. Outcomes included kinesiophobia, pain catastrophizing, self-efficacy, exercise adherence, pain intensity, knee range of motion, and quadriceps strength. Assessments were performed by blinded evaluators before and after the intervention.

**Results:**

Both groups showed significant improvements in all outcome measures (*p* < 0.05), with the motor imagery group achieving significantly greater gains in psychological variables, pain reduction, and physical function compared with the control group (*p* < 0.05).

**Conclusion:**

Motor imagery is an effective adjunct to early rehabilitation after total knee arthroplasty. Its use enhances psychological resilience, supports adherence, and accelerates functional recovery, indicating its value in comprehensive, multidimensional rehabilitation strategies.

## LAY ABSTRACT

This study tested a mental training technique called motor imagery in people recovering from knee replacement surgery. Motor imagery helps patients imagine movements before they actually do them. Forty patients were divided into 2 groups: 1 followed standard rehabilitation, and the other used motor imagery along with the same rehabilitation. The goal was to reduce fear of movement, known as kinesiophobia, improve mental strength, reduce pain, and support physical recovery. After 2 weeks, both groups improved, but those who used motor imagery showed better results in pain relief, knee movement, leg strength, and motivation to stick with exercise. This shows that thinking about movement, even without doing it, can help the body heal faster and better. Motor imagery is easy to add to rehabilitation and does not require special equipment, making it useful for hospitals and clinics. It may also help people who are too afraid or in too much pain to move early after surgery.

Osteoarthritis is the most common joint disorder among individuals over the age of 60 worldwide, affecting approximately 10% of men and 18% of women (1). It frequently occurs in weight-bearing joints, particularly the knee (2). The prevalence and incidence of knee osteoarthritis (KOA) increase markedly between the ages of 50 and 70 (3, 4). When KOA becomes chronic, it leads to pain, joint deformities, and muscle weakness, resulting in impaired function, reduced ability to perform daily activities, and decreased quality of life (5–8). For patients with end-stage KOA unresponsive to conservative treatment, total knee arthroplasty (TKA) is the standard surgical intervention (9–11). KOA presents a growing socioeconomic burden due to productivity loss, increased healthcare costs, and reduced quality of life (12).

Although TKA is effective, patients in the acute postoperative phase commonly experience pain, reduced range of motion (ROM), and quadriceps weakness (13). This weakness can further restrict ROM, impair gait, and reduce functional capacity, thereby hindering daily activities (14–16). Early rehabilitation is essential to reduce pain and swelling, restore ROM and muscle strength, and improve function while minimizing disability (17). Standard physical therapy includes cryotherapy, neuromuscular stimulation, continuous passive motion, ROM exercises, resistance training, and weight-bearing activities (18–21). However, due to pain and hypersensitivity, many patients voluntarily restrict movement during early rehabilitation (10). This may develop into kinesiophobia, an irrational fear of movement, which contributes to pain persistence and delays in recovery, ultimately limiting rehabilitation effectiveness (22, 23). Kinesiophobia can reduce exercise adherence (EA) and impede improvement (22, 24–26).

Patients with high kinesiophobia often avoid postoperative exercise, increasing the risk of prolonged pain and weakness (23). Kinesiophobia has a greater impact on functional limitations and disability than on the actual intensity of pain, and this fear persists even after pain has subsided, acting as a major factor delaying recovery (27). In particular, this phenomenon is commonly observed in postoperative patients and is associated with reduced active ROM as well as sustained negative effects on long-term outcomes (28). Pain catastrophizing (PC) and self-efficacy (SE) are closely linked with EA. High PC promotes avoidance, whereas high SE fosters exercise engagement and recovery (29, 30). Motor imagery (MI), a mental simulation of movement, is gaining interest as a cognitive rehabilitation strategy. Simulated motor strategies may even be more effective than physical movement (29, 30). MI, a mental simulation of movement, is gaining interest as a cognitive rehabilitation strategy (31, 32).

Motor imagery activates motor areas of the nervous system without actual execution, aiming to enhance motor performance (33–36). Studies show that MI training induces neural patterns similar to actual movement, especially in the premotor cortex, cerebellum, and supplementary motor areas. This similarity has also been confirmed by functional magnetic resonance imaging (37–39). MI training, which applies the theoretical mechanisms of MI to structured rehabilitation training, connects with descending pathways, enhances cortical motor planning, and promotes neuroplasticity, thereby facilitating muscle activation and synchronization (36, 40–42).

Mizner et al. (43) reported that quadriceps strength (QS) loss following TKA is not solely attributable to muscle atrophy but is largely due to failure of voluntary activation. Similarly, Paravlic et al. (44) demonstrated that maximal voluntary strength of the quadriceps decreases sharply immediately after surgery, with the greatest decline occurring on the third postoperative day. These findings suggest that interventions initiated within the first 48 h – prior to the peak of strength loss – represent a critical window to prevent the progression of arthrogenic muscle inhibition and to promote functional recovery. Furthermore, psychological and cognitive factors, including kinesiophobia, have been shown to negatively influence functional outcomes for up to 1 year after TKA (28). Although complementary strategies to enhance early TKA rehabilitation are increasingly emphasized (42), current approaches still focus mainly on physical recovery, with limited attention to psychological factors (45). This study aims to examine the effects of a rehabilitation programme incorporating MI training on acute-phase TKA patients with high kinesiophobia, assessing its impact on psychological variables, EA, pain, and physical function.

## METHODS

### Participants and design

This single-blind, randomized controlled trial was conducted at K Hospital in Seoul, South Korea, to evaluate the effectiveness of an acute-phase rehabilitation programme incorporating MI training in patients who had undergone total knee arthroplasty (TKA) and exhibited symptoms of kinesiophobia. Forty patients diagnosed with end-stage knee osteoarthritis (KOA; Kellgren–Lawrence grade ≥ 3) by an orthopedic specialist were recruited (46). Participants were required to have received a prescription for acute-phase physical therapy within 48 hs postoperatively (10) and to score ≥ 37 on the Tampa Scale of Kinesiophobia (TSK), indicating high levels of kinesiophobia (47).

Exclusion criteria included prior knee surgery within 1 year before TKA, revision surgery, bilateral TKA, postoperative complications such as infection, inflammation, or fracture (10, 13), neurological disorders affecting gait (e.g., stroke, Parkinson’s disease), cognitive impairment (Mini-Mental State Examination score < 24), and severe lumbar or hip conditions that could interfere with participation.

After completing baseline assessments, participants were randomly assigned to either the experimental group (EG), which participated in an acute-phase rehabilitation programme with MI training, or the control group (CG), which received the same programme without MI training. Randomization was conducted by a research assistant using a computer-generated algorithm, and allocation concealment was maintained to ensure group assignments were not known in advance. Simple randomization without restrictions such as blocking or stratification was applied. All participants and outcome assessors were blinded to the group allocation. For clinical reasons, group allocation information was provided to the therapists administering the interventions.

The intervention was provided once daily, 5 times per week, over a 2-week period. One therapist delivered the intervention to both groups following a standardized protocol, and a blinded assessor, uninvolved in the intervention, conducted all outcome evaluations using the same measures at baseline and post-intervention. To ensure study continuity and address potential staff absence, 1 alternative therapist and 1 alternative assessor were designated in advance. All personnel involved in the study were trained and familiarized with the protocol.

Before enrolment, the purpose, procedures, and expected outcomes were fully explained to all participants, and written informed consent was voluntarily obtained. This study was conducted in accordance with the ethical principles outlined in the Declaration of Helsinki and was approved by the Institutional Review Board of Sahmyook University (approval number: SYU 2024-02-018-001). Also, this study was retrospectively registered on CRIS (ID: KCT0010422) following the initiation of participant enrolment, in order to ensure transparency and adherence to international reporting standards.

### Intervention

Both groups underwent interventions based on the same standardized acute-phase rehabilitation exercise programme. The EG performed standard rehabilitation exercises in combination with MI training, whereas the CG performed standard rehabilitation exercises without MI training. The intervention was provided once daily, 5 times per week, for 2 weeks postoperatively, with each session lasting approximately 30 min.

**Fig. 1:**
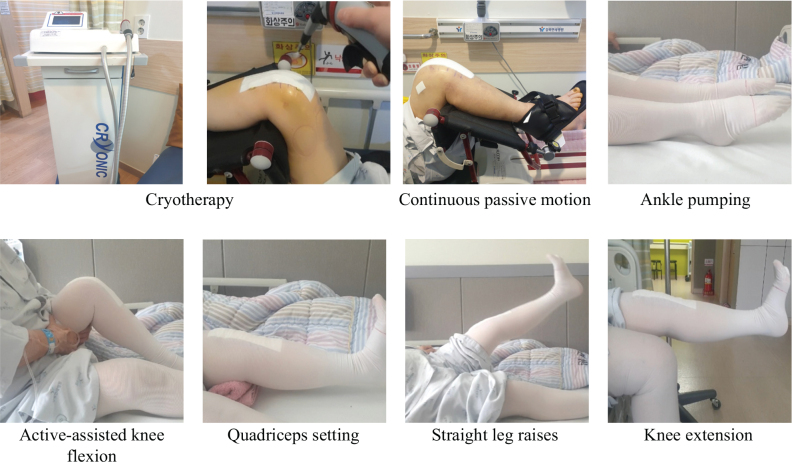
Rehabilitation programme.

The aim of the acute-phase rehabilitation programme, initiated 48 h after TKA, was to reduce pain and swelling and improve joint ROM and muscular function (48). Based on the protocol established by Briones-Cantero et al. (10), the programme included cryotherapy, CPM, ankle pumping, active-assisted knee flexion, quadriceps setting, straight leg raises, and knee extension exercises (Fig. 1). All participants performed each exercise for 10 repetitions, holding each movement for 5 s, with a 30-s rest between exercises to prevent fatigue accumulation. All sessions were conducted by the same physical therapist, following a standardized protocol to ensure consistency across sessions.

Motor imagery training was conducted prior to the standard rehabilitation exercises to reduce psychological resistance to movement and enhance neural readiness. This allowed participants to mentally simulate each movement in advance. MI training was applied to 5 rehabilitation exercises: ankle pumping, active-assisted knee flexion, quadriceps setting, straight leg raises, and knee extension. The intervention was structured as follows. Step 1: The participants performed each of the 5 rehabilitation exercises 10 times using their non-operated limb, while the researcher recorded the movements using a smartphone. Step 2: The participants watched recorded videos of their performance and simultaneously imagined performing the same movements as with their operated limb. Step 3: The therapist provided still images of each exercise performed with maximum ROM using the non-operated limb. These visual references were used to vividly recall sensations such as muscle contraction, joint positioning, and shifts in the centre of gravity. Step 4: The participants performed each exercise 10 times with the operated limb to reinforce the neural linkage between imagery and actual movement.

The motor imagery training protocol was designed by integrating the principles of passive, active, and combined MI. The participants were initially guided through visual stimuli (videos and images) to develop external visual awareness of their movements. This gradually transitioned into internal kinaesthetic imagery, culminating in the actual execution of the movements to enhance sensorimotor integration. During the training, the therapist provided detailed explanations of the key sensory aspects of movement – such as the sensation of shifting weight, feeling of increasing joint ROM, and perception of muscle contraction or resistance – allowing participants to internalize realistic motor sensations during imagery practice. The training was tailored to each participant’s functional capacity. The researcher monitored participant responses, provided additional video resources when necessary, and gave feedback accordingly. The participants completed a daily training log, and the researcher checked their adherence and offered supplementary instructions when required.

### Outcome measures

The effects of the intervention were evaluated based on the following outcome measures: kinesiophobia, psychological variables (PC and SE), EA, pain, and physical function (knee ROM and QS). All variables were assessed under the same conditions before and after the intervention.

Kinesiophobia was assessed using the TSK-17, a 17-item self-reported questionnaire that measures fear of movement and physical activity (49, 50). Higher scores indicate greater levels of kinesiophobia (47, 51). The scale has demonstrated high reliability, with an intraclass correlation coefficient of 0.96 (47) and a Cronbach’s alpha of 0.84 (52).

Pain catastrophizing, defined as an exaggerated negative cognitive response to actual or anticipated pain (23, 53, 54), was measured using the Korean version of the Pain Catastrophizing Scale (PCS) (54). Higher scores reflect higher levels of catastrophizing (55). The scale has demonstrated good reliability with a test–retest reliability of 0.79 and a Cronbach’s alpha of 0.93 (56).

Self-efficacy refers to an individual’s confidence and belief in their ability to perform behaviours related to disease management or functional tasks (57). The Korean version of the Arthritis Self-Efficacy Scale, originally developed by Lorig and Holman (58), was used. This version was culturally adapted by Oh and Kim (59) for Korean patients with degenerative arthritis. The scale consists of 10 items modified from the original 20-item version. Each item was scored in increments of 10 points, with 10 indicating no confidence and 100 indicating complete confidence. The total score was calculated by summing the scores of the 10 items, yielding a range of 100 to 1,000 points. Higher scores indicate greater SE. The intra-rater reliability of this instrument has been reported as 0.81, and the internal consistency was Cronbach’s α = 0.81 (60).

Exercise adherence, defined as the extent to which individuals consistently follow prescribed exercise programmes (61), was assessed using the Korean Exercise Adherence Questionnaire (EAQ) developed by Choi (62) based on theoretical frameworks including Ajzen’s theory of planned behaviour, the Corbin and Lindsey model, and Stebbins’ leisure theory (63–67). This 17-item questionnaire consists of 5 subcomponents and uses a 4-point Likert scale ranging from “strongly disagree” to “strongly agree”. Higher scores indicate greater EA. The subscale reliability coefficients ranged from 0.741 to 0.787 (62).

Pain was assessed using a Visual Analogue Scale (VAS). Participants were instructed to indicate their current level of knee pain on a 100 mm horizontal line without markings, where 0 mm represented “no pain” and 100 mm represented “worst imaginable pain” (10). The marked point was then measured using a ruler to quantify pain intensity. The VAS is a reliable and valid tool for quantifying pain following TKA (68), with a reported test–retest reliability of 0.62 and validity of 0.75 (69).

Physical function was evaluated based on knee ROM and QS. The ROM was measured using a smartphone application (Smart Protractor 1.5.16a; Smart Tools, Korea, 2010). The intra-rater and inter-rater reliabilities were reported as 0.81 and 0.79, respectively (70).

Isometric QS was assessed using the K-Pull device (K Force Pro; KINVENT, Montpellier, France, 2022). The participants were seated with 90° hip flexion and 45° knee extension and instructed to perform maximal knee extension contractions 3 times, with 10-sd rest intervals between trials. The average of the 3 trials was used for the analysis. The inter-rater reliability of this method was reported as 0.93 (71).

### Sample size calculation

The sample size was determined by calculating the effect size for the TSK difference between the experimental and control groups reported in the prior acute post-TKA study by Cai et al. (72), which enrolled patients with high kinesiophobia (TSK ≥ 7). G*Power software (version 3.1.2; Franz Faul, University of Kiel, Kiel, Germany) was used, and using an effect size of 0.97 obtained from the prior study with α = 0.05 and power (1−β) = 0.80, the required sample size was calculated to be 36. Considering an expected 10% attrition, a total of 40 participants was recruited.

### Data analysis

All statistical analyses were performed using IBM SPSS Statistics version 29.0 (IBM Corp, Armonk, NY, USA, 2023). The normality of all data was assessed using the Shapiro–Wilk test. To examine baseline homogeneity between groups, χ^2^ and independent *t*-tests were conducted. Paired *t*-tests were used to compare pre- and post-intervention changes within each group, and independent *t*-tests were employed to compare differences between groups. The level of statistical significance was set at *p* < 0.05 for all analyses.

## RESULTS

### General characteristics

The first participant was enrolled on 20 April 2024, and recruitment was completed on 13 January 2025. Forty participants were enrolled, with 20 assigned to the EG and 20 to the CG. All participants completed the study without any dropouts or exclusions. The study was completed as planned without early termination. No statistically significant differences were observed between the groups in the baseline general characteristics (age, height, weight, and side of surgery) or the TSK-17 scores (Table I). No harms or unintended effects were observed in either group during the intervention period.

**Table I:** General characteristics

Item	EG (*n* = 20)	CG (*n* = 20)	*χ^2^/t*(*p*)
Sex (male/female)	6/14	7/13	0.114^a^(0.736)
Surgical site (right/left)	12/8	11/9	0.102 (0.749)
Age, years, mean ± SD	69.30 ± 2.57	69.80 ± 2.09	–0.674^b^ (0.505)
Height, cm, mean ± SD	163.48 ± 6.33	164.87 ± 5.48	–0.745 (0.461)
Weight , kg, mean ± SD	62.81 ± 6.39	64.53 ± 5.93	–0.879 (0.385)
TSK-17, pts, mean ± SD	45.35 ± 1.63	44.95 ± 1.50	0.806 (0.425)

a χ^2^ test;

b independent *t*-test.

SD: standard deviation; TSK: Tampa Scale of Kinesiophobia 17; EG: experimental group; CG: control group.

### Changes in kinesiophobia (TSK)

**Fig. 2:**
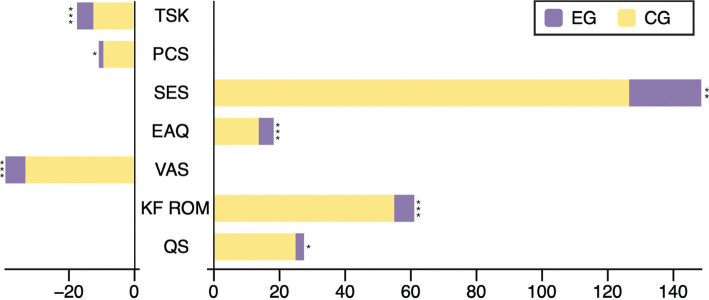
Between-group comparison of changes in outcome measures. EG: experimental group; CG: control group; TSK: Tampa Scale for Kinesiophobia; PCS: Pain Catastrophizing Scale; SES: Self-Efficacy Scale; EAQ: Exercise Adherence Questionnaire; VAS: Visual Analogue Scale; KF ROM: knee flexion range of motion; QS: quadriceps strength. *p < 0.05, **p < 0.01, ***p < 0.001.

Both groups showed a significant reduction in kinesiophobia scores (*p* < 0.05). In the EG, the score significantly decreased from 45.35 ± 5.21 at pre-test to 27.80 ± 4.90 at post-test (*p* = 0.001). In the CG, the score also significantly decreased from 44.95 ± 5.08 to 32.40 ± 5.22 (*p* = 0.001). When comparing the amount of change between groups, the EG showed a greater score reduction (17.55 ± 2.35) than in the CG (12.55 ± 2.60) (*p* = 0.001) (Table II, Fig. 2).

**Table II:** Within-group and between-group differences in outcome measures

Variable	Group	Pre, mean ± SD	Post, mean ± SD	Change, mean ± SD	*t*(*p*)	95% CI^a^	*t*(*p*)^b^
TSK (pts)	EG	45.35 ± 1.63	27.80 ± 1.24	–17.55 ± 2.35	33.395(0.001^*^)	−5.00 (−6.59, −3.41)	–6.373(0.001^*^)
	CG	44.95 ± 1.50	32.40 ± 2.37	–12.55 ± 2.60	21.544(0.001^*^)		
PCS (pts)	EG	35.70 ± 2.63	24.80 ± 1.32	–10.90 ± 2.38	20.465(0.001^*^)	−1.40 (−2.77, −0.04)	–2.076(0.045^*^)
	CG	35.55 ± 2.35	26.05 ± 2.28	–9.50 ± 1.85	22.970(0.001^*^)		
SES (pts)	EG	174.00 ± 14.29	322.50 ± 20.74	148.50 ± 21.83	–30.421(0.001^*^)	22.00 (8.34, 35.66)	3.260(0.002^*^)
	CG	175.50 ± 13.56	302.00 ± 20.92	126.50 ± 20.84	–27.141(0.001^*^)		
EAQ (pts)	EG	29.00 ± 1.33	47.20 ± 1.36	18.20 ± 1.98	–40.912(0.001^*^)	4.55 (3.30, 5.80)	7.347(0.001^*^)
	CG	28.55 ± 1.23	42.20 ± 1.36	13.65 ± 1.92	–31.679(0.001^*^)		
VAS (mm)	EG	78.25 ± 4.10	38.90 ± 1.71	–39.35 ± 4.62	38.043(0.001^*^)	−6.10 (−8.95, −3.25)	–4.335(0.001^*^)
	CG	76.05 ± 4.18	42.80 ± 2.28	–33.25 ± 4.26	34.858(0.001^*^)		
KF ROM (°)	EG	52.25 ± 1.16	113.30 ± 2.25	61.05 ± 2.76	–98.848(0.001^*^)	6.10 (4.51, 7.70)	7.742(0.001^*^)
	CG	52.50 ± 1.46	107.45 ± 2.37	54.95 ± 2.18	–112.320(0.001^*^)		
QS (N)	EG	110.19 ± 2.85	137.66 ± 2.55	27.46 ± 4.73	–25.950(0.001^*^)	2.60 (0.05, 5.16)	2.064(0.046^*^)
	CG	110.20 ± 3.00	135.06 ± 0.97	24.86 ± 3.06	–36.276(0.001^*^)		

a 95% CI for the between-group difference in change scores;

b *p*-value for the difference in pre–post change between groups.

SD: standard deviation; TSK: Tampa Scale for Kinesiophobia; PCS: Pain Catastrophizing Scale; SES: Self-Efficacy Scale; EAQ: Exercise Adherence Questionnaire; VAS: Visual Analogue Scale; KF ROM: knee flexion range of motion; QS: quadriceps strength; N: Newton; EG: experimental group; CG: control group.

* *p* < 0.05.

### Changes in pain catastrophizing (PCS)

Pain catastrophizing scores significantly decreased in both groups after the intervention (*p* < 0.05). The EG showed a significant reduction from 35.70 to 24.80 (*p* = 0.001), and the CG showed a decrease from 35.55 to 26.05 (*p* = 0.001). The amount of change was greater in the EG (–10.90) than in the CG (–9.50) (*p* = 0.045).

### Changes in self-efficacy (SES)

Self-efficacy scores significantly increased in both groups after the intervention (*p* < 0.05). In the EG, the score improved from 174.00 to 322.50 (*p* = 0.001), while in the CG, it increased from 175.50 to 302.00 (*p* = 0.001). The EG showed a greater increase (148.50) than in the CG (126.50) (*p* = 0.002).

### Changes in Exercise Adherence Questionnaire (EAQ)

Exercise adherence scores significantly improved in both groups after the intervention (*p* < 0.05). In the EG, the score increased from 29.00 to 47.20 (*p* = 0.001), while in the CG, it improved from 28.55 to 42.20 (*p* = 0.001). Between-group comparison of the change in scores revealed a greater increase in the EG (18.20) than in the CG (13.65) (*p* = 0.001).

### Changes in pain (VAS)

Pain scores significantly decreased in both groups (*p* < 0.05). In the EG, the score was reduced from 78.25 mm to 38.90 mm (*p* ≤ 0.001), and in the CG, from 76.05 mm to 42.80 mm (*p* = 0.001). The change in pain intensity was significantly greater in the EG (−39.35 mm) than in the CG (−33.25 mm) (*p* = 0.001).

### Changes in knee flexion ROM (KF ROM)

Both groups demonstrated a significant improvement in knee flexion ROM (*p* < 0.05). The ROM in the EG increased from 52.25° to 113.30° (*p* = 0.001), while that in the CG increased from 52.50° to 107.45° (*p* = 0.001). The EG showed a greater improvement (61.05°) than in the CG (54.95°) (*p* = 0.001).

### Changes in quadriceps strength (QS)

Quadriceps Strength significantly improved in both groups (*p* < 0.05). The EG showed an increase from 110.19 N to 137.66 N (*p* = 0.001), and the CG from 110.20 N to 135.06 N (*p* = 0.001). The extent of improvement was significantly greater in the EG (27.46 N) than in the CG (24.86 N) (*p* = 0.046).

## DISCUSSION

In this study, we aimed to compare the effects of an acute-phase rehabilitation programme, with and without MI training, in patients with high kinesiophobia after TKA. The results demonstrated significant improvements in both groups across all outcome variables, including kinesiophobia, psychological factors, EA, pain, and physical function (*p* < 0.05). Notably, the EG that underwent MI training showed significantly greater improvement than the CG (*p* < 0.05). These findings suggest that MI can serve as an effective intervention during acute rehabilitation by enhancing physical recovery and modulating psychological factors that influence rehabilitation participation. MI is a cognitive technique involving the mental simulation of movements without actual physical execution. It activates multiple cortical regions in a manner similar to real movement, thereby promoting neuroplasticity and positively influencing the central nervous system (73, 74). The significantly greater reduction in kinesiophobia and PC observed in the EG supports the clinical applicability of these neurological mechanisms in real-world rehabilitation settings.

Furthermore, MI activates neural circuits involved in emotional regulation and fear perception by stimulating the prefrontal cortex and midbrain–limbic pathways (75, 76). These activations may facilitate not only the regulation of negative emotions but also the modulation of fear-avoidance behaviours, thereby creating a more favourable psychological environment for active participation in rehabilitation. This mechanism may contribute to emotional stability and a reduction in fear avoidance behaviours. In the present study, the EG showed significantly greater reductions in kinesiophobia and PC than in the CG, suggesting that MI facilitated psychological improvement and enhanced patient engagement in rehabilitation. Similar results have been reported in previous studies. Moseley (77) demonstrated that MI activates the neural networks responsible for emotional and behavioural regulation and Parsons (78) identified neurological mechanisms related to the modulation of pain perception in the brain. Additionally, reductions in kinesiophobia and PC following MI have been observed in patients recovering from spinal surgery (79) and in those with chronic shoulder pain (80). However, most studies have focused on regions subjected to indirect weight-bearing, such as the lumbar spine and shoulders. Evidence regarding the joints exposed to direct weight-bearing during gait, such as the knee, remains scarce. In patients undergoing TKA, chronic pain and functional impairment often precede surgery, and postoperative psychological distress and exercise avoidance are common. These factors raise the possibility that the effectiveness of MI may vary depending on the anatomical region involved. Thus, this study is clinically significant because we empirically investigated the impact of MI on psychological and physical recovery during acute-phase rehabilitation in a directly weight-bearing joint: the knee.

Kinesiophobia and PC are the major psychological factors limiting rehabilitation exercise participation in patients after TKA, and SE plays a key mediating and moderating role in these mechanisms (23). In the present study, the EG showed a significantly greater increase in SE than the CG, suggesting that MI effectively enhances the patients’ confidence and belief in their ability to perform physical tasks. This finding aligns with those of previous studies, indicating that higher SE is associated with more active engagement in rehabilitation and better long-term functional recovery (81, 82).

Motor imagery provides continuous cognitive stimulation of the cerebral cortex, and repeated imagery training can help strengthen patients’ confidence in movement execution (83, 84). The significantly higher EA observed in the EG suggests that MI reinforces motivation and consistent exercise performance during TKA rehabilitation.

Motor imagery also plays an important role in reducing pain and improving physical function. It activates the motor and premotor cortices, eliciting neural responses similar to actual movement (85, 86), and has been reported to reorganize the sensorimotor network, thereby alleviating pain perception (87, 88). In the current study, the EG showed a greater reduction in pain than the control group (*p* < 0.05), with a decrease of 39.35 mm on the VAS, exceeding the minimal clinically important difference of 30 mm (89).

Regarding ROM and QS, the EG demonstrated significantly greater improvement than the CG. Specifically, knee flexion ROM reached 113.30°, surpassing the clinically meaningful threshold of 100–110° (90). QS increased by 27.46 N, exceeding the minimal clinically important change of 21.89 N (91). These findings suggest that MI did not directly increase muscle strength but rather facilitated greater participation in exercise by reducing kinesiophobia and PC, thereby promoting strength gains and functional recovery. These results are consistent with those of previous studies by Zhou et al. (23), Araya-Quintanilla et al. (80), and Louw et al. (79).

In this study, we empirically confirmed that MI positively affects psychological variables, such as kinesiophobia, PC, SE, and EA, and physical recovery outcomes, including pain, ROM, and muscle strength. In particular, MI interventions during the acute rehabilitation phase have demonstrated clinical utility by reducing psychological resistance and avoidance behaviours, enhancing adherence to rehabilitation programmes, and ultimately promoting functional recovery. These findings suggest the need for a multidimensional rehabilitation strategy that integrates psychological and cognitive interventions in addition to conventional physical therapy. Such a strategy can provide a theoretical and clinical foundation for the expanded application of MI across various joint disorders and postoperative recovery contexts.

Following TKA, periarticular inflammation, intra-articular effusion, and pain stimulate mechanoreceptors and nociceptors around the joint, leading to a reduction in the excitability of the α-motoneuron pool at the spinal level (92). This results in arthrogenic muscle inhibition, a condition in which patients are unable to fully activate the quadriceps despite voluntary effort, thereby causing a marked decline in muscle strength (16, 43). In addition, kinesiophobia, defined as the fear of movement and reinjury after surgery, hinders functional recovery throughout the rehabilitation process (93). Kinesiophobia is not merely an expression of anxiety; rather, it amplifies pain perception through pain catastrophizing and serves as a critical barrier to functional improvement (94). Indeed, it has been reported that kinesiophobia may negatively influence functional outcomes for up to 12 months postoperatively (28). According to Vlaeyen and Linton’s (94) fear-avoidance model, pain catastrophizing, and kinesiophobia reinforce avoidance behaviours and impede functional recovery, while Bandura’s (81) self-efficacy theory posits that SE is a key determinant of initiating and maintaining behaviour, including adherence to exercise. From this integrative perspective, MI may provide a safe and pain-free experience of movement, thereby alleviating both the neurological inhibition associated with arthrogenic muscle inhibition and the psychological inhibition associated with kinesiophobia. This process may enhance SE, promote EA, and ultimately contribute to improvements in function and quadriceps strength. Therefore, in patients with TKA who present with kinesiophobia, both the neurophysiological activation achieved through MI and the psychological dimensions of rehabilitation – kinesiophobia, SE, and EA – should be considered together (94, 95). Paravlic et al. (44) reported that QS declines sharply immediately after TKA and reaches its maximum reduction within 3 days. In light of the favourable effects demonstrated in the present study, the application of MI-based early rehabilitation within 48 h after surgery appears to represent not only a critical time window but also an effective strategy for minimizing strength loss and promoting early functional recovery.

This study did not include follow-up assessments. Therefore, the long-term effects could not be directly confirmed. However, previous research has shown that kinesiophobia can negatively affect ROM and muscle strength for up to 1 year after TKA (28), suggesting that MI may also have the potential to contribute to long-term functional improvement. Nevertheless, future studies with extended follow-up are required to verify this possibility. The average age of the participants was under 70, which limits the generalizability of the results to older populations. Additionally, as daily activities during hospitalization were not fully controlled, the potential influence of external factors cannot be eliminated. The findings of this study are specific to patients with high kinesiophobia and should be interpreted with caution when considering generalization to the broader TKA population. Future research should consider broader age groups and incorporate long-term analyses with a tighter control of lifestyle variables. Previous research has mainly focused on non-weight-bearing joints. However, our study is novel in that it applied MI to a weight-bearing joint, such as the knee, thereby suggesting the potential for expanding its use in orthopaedic rehabilitation.

In conclusion, in this study we examined the effects of a rehabilitation programme incorporating MI on psychological factors (kinesiophobia, PC, SE, and EA) and physical function (pain, ROM, and QS) in acute-phase patients with high levels of kinesiophobia following TKA. Both groups demonstrated significant improvements, with the EG showing significantly greater changes in all variables (*p* < 0.05). These findings empirically support the idea that MI, when integrated with conventional physical rehabilitation, is effective in alleviating psychological resistance and enhancing functional recovery in acute-stage TKA patients. This suggests the clinical potential of MI as a multidimensional rehabilitation strategy applicable to various joint disorders and postoperative recovery settings.
